# Prevalence and Concomitants of Arthritis in the Elderly in Rio Grande do Sul, Brazil

**DOI:** 10.1371/journal.pone.0045418

**Published:** 2012-09-28

**Authors:** Sergio L. Blay, Gerda G. Fillenbaum, Sergio B. Andreoli, Fábio L. Gastal

**Affiliations:** 1 Department of Psychiatry, Federal University of São Paulo, São Paulo, Brazil (Escola Paulista de Medicina – UNIFESP); 2 Center for the Study of Aging and Human Development, Duke University Medical Center, Durham, North Carolina, United States of America; 3 Geriatric Research, Education and Clinical Center, Veterans Administration Medical Center, Durham, North Carolina, United States of America; 4 Project Scientific Committee Medical Director, Sistema de Saúde Mãe de Deus, Porto Alegre, Brazil; Federal University of Rio de Janeiro, Brazil

## Abstract

**Objectives:**

Information on the prevalence and concomitants of arthritis in developing countries is sparse. It is unclear whether they are comparable to findings in developed countries. To ascertain the prevalence, demographic characteristics, and health-related concomitants of arthritis in older persons in the southern state of Rio Grande do Sul, Brazil, a middle income country.

**Methods:**

The state of Rio Grande do Sul, Brazil, was subdivided into nine regions. Stratified random sampling was used to identify 880 community residents age ≥60 years in each region. One region with suspect data was excluded. Of 7040 community residents contacted in eight regions, 6963 participated (1.1% refusal rate). In 1995, trained, monitored interviewers, using structured questionnaires, conducted in-home interviews gathering information on demographic characteristics (age, sex, race/ethnicity, education, income, living arrangements, employment status), health behaviors (physical activity, tobacco use, social activity), functional limitations, depression, and 15 self-reported health conditions, including arthritis. Data were analyzed using descriptive statistics and logistic regression.

**Results:**

Arthritis, reported by 43% of the sample, was more prevalent in women, among the less educated, those with lower income, and higher age. Severity, but not prevalence, differed by race/ethnicity. Controlled analyses indicated significant association with female gender, lower education, and less social activity. Arthritis was associated with reduced odds of stroke, but increased odds of hypertension, varicosities, bronchitis, renal problems, headache, gastrointestinal disorders, and depression. Arthritis was not significantly associated with age or functional limitations, and associations did not differ by gender.

**Conclusions:**

The prevalence, demographic and health characteristics associated with self-reported arthritis in this southern state in Brazil are similar to findings elsewhere in Brazil, and in developed countries.

## Introduction

Arthritis (the term used here to encompass arthritic and rheumatic diseases), is a highly prevalent, painful, chronic condition common in older people, and often associated with functional limitations that can be severely disabling [1]–[5].

Numerous nationally representative [6]–[17], as well as geographically-specific studies [18]–[29], on the prevalence, concomitants, and impact of arthritis have been carried out, but information on the prevalence of arthritis from the South American continent, particularly in the older population, is conflicting. Specifically, reports from well-designed, nationally representative and locally representative studies report rates ranging from 12.2% to 51.9% [17], [23]–[27]. Differences seem to reflect how arthritis is defined, how enquiry is phrased, and the time frame covered. While functional limitations have been a matter of concern, attention has been paid to the concomitant presence of a very limited number of other chronic health conditions which may be associated with arthritis and which themselves are associated with functional limitations.

Population-based studies in developed countries indicate that 50% or more of persons age ≥65 have self-reported or doctor-diagnosed arthritis. Uniformly, arthritis has a higher prevalence in women, increases with age, and is associated with functional limitations. In the U.S., arthritis occurs comparably in Whites and African Americans [30], information for Brazil does not seem to have been reported.

Reported risk factors for arthritis, in addition to gender and age, include genetics, smoking, occupational hazards associated with biomechanical stresses, trauma, obesity, and possibly, less education [1], [10], [31], [32]. Arthritis has been found to be associated with depression and other psychiatric disorders [14], [31], [32] and chronic conditions [11], [33], [34]. It is currently unclear whether rates and associations found elsewhere hold for countries in Latin America. As a step in addressing this question, we have examined data from a representative statewide sample of nearly 7,000 community residents age ≥60 years in the southern part of Brazil. Here we report the prevalence and concomitants of arthritis (demographic characteristics, health behaviors, functional limitations, health conditions) in this state. Our findings provide additional information on these matters for a middle income country.

## Methods

### Sample

Selection of community residents 60 years of age or older, was based on a multistage, stratified random-sampling procedure in the state of Rio Grande do Sul, in the southern region of Brazil. As previously reported [35], this region has a largely agro-industrial economy, and is populated primarily by descendents of European immigrants. The state was divided into nine homogeneous areas. The first step was to stratify the 333 municipalities into five categories according to basic economic activity and number of inhabitants. The proportion of each category in each homogeneous zone was calculated, and the number of subjects in each stratum needed to obtain a representative proportion of elderly community residents was determined. Second, the municipalities were randomly selected proportionally in each stratum. The third step was to obtain a random sample of urban census areas for each municipality as supplied by the Brazil Geography and Statistics Institute (Instituto Brasileiro de Geografia e Estatística). Fourth, to get a sample of private households from each of these census areas, a block was randomly selected and every eighth house was systematically visited in person by the interviewer. If a household had more than one age-eligible person, the respondent was randomly selected. Houses with no eligible person were replaced by the next neighbor. The sample was representative of community residents of this age group in the state.

Data were gathered in 1995 by specifically trained, closely monitored interviewers, using structured face-to-face household surveys with 880 residents of each region. Data entry problems were identified in one region, which was then excluded from analysis. In the remaining eight regions, 7040 persons were approached. No proxy information was collected. Only 77 persons (1.1%) did not take part in the assessment, primarily refusals, yielding an overall response rate of 99% (N = 6963). The study was approved by the Ethics Committee of the Federal University of São Paulo. Participants gave oral consent.

### Information obtained

The information sought included sociodemographic characteristics, health behaviors, functional limitations, and physical and mental health conditions.

Sociodemographic characteristics included gender, age (coded as 60–64, 65–69, 70–74, 75–79, ≥80 years of age), education (<4 years, ≥4 years), income (<$US200/month [low income] *vs*. ≥$US200/month [higher income]), race/ethnicity (White, Afro-Brazilian, other), living arrangement (live alone *vs*. living with others), and whether currently employed.

Health behaviors included whether physically active (‘In the last three months did you practice any kind of regular physical activity?’ Report of once or more a week was coded as ‘Yes’; report of ‘No’ or ‘Don’t know’ was coded as ‘No’), whether used tobacco (yes/no), and current participation in social activities (participation in any formal association on a list that included cultural, sports, recreational, religious, charitable/aid providing, community, union, political, and ‘other’ associations was coded as ‘Yes’, report of ‘No’ or ‘Don't know’ was coded as ‘No’.).

Functional limitations were measured on a 5-item unidimensional scale that included instrumental and basic activities of daily living [35]. Each item was scored dichotomously (need help vs. able to perform independently). The number of items with which help was needed was summed (range 0–5), and categorized as 0, 1–2, or 3 or more items with limitations.

#### Physical and mental health

The specific question on arthritis (translated) was: In the last six months did you seek treatment because of problems with rheumatism in your joints or arthritis? (yes/no). Enquiry into other health conditions for which treatment had been sought in the previous six months included hypertension, heart problems, stroke, varicosities, diabetes, back problems, osteoporosis, bronchitis, pneumonia, urinary tract infection, renal problems, dermatologic problems, headaches (in the previous week), and gastro-intestinal problems. Since two thirds of those with back problems also reported arthritis (and vice versa), back problems were not included in the list of health conditions further examined to avoid the possibility that for the majority of sufferers, back problems represented a symptom of arthritis, and not a separate condition. The presence of depression within the previous 30 days was determined by the six-item Short Psychiatric Evaluation Schedule (Short-SPES), validated for the older Brazilian population [36], [37]. The total score (potential scoring range 0–6) was categorized as 0 (no depressive symptoms), 1, 2, 3 or more depressive symptoms.

### Statistical analyses

Descriptive statistics were used to characterize the data, and to calculate bivariate associations with arthritis. Significant bivariate findings (p≤0.01, with the exception of age which was considered too important to omit) were entered into logistic regression analyses to examine the association with arthritis of sociodemographic characteristics, health behaviors, and functional limitations. The variables significant in this analysis were then entered into the final analysis, which included all health conditions. We report odds ratios and 95% confidence intervals, but because of the sample size and number of analyses p≤.01 was used to determine significance. To determine whether statistically significant gender differences were present, the final model was re-run for the total sample with gender interaction terms for all variables, and the same criterion to identify significance.

## Results

Approximately two thirds of the sample were female, had low education and income, and just over half were under 70 years of age. The sample was preponderantly White (84%) (Table 1). Just over a third participated in some kind of physical or social activity, only 14% were currently employed, or used tobacco. Few lived alone, nearly 40% had some functional limitation.

**Table 1:** Sample characteristics by presence or absence of self-reported arthritis.

		Arthritis					
	Total sample N (6963)	Absent (N = 3961)	Present (N = 3002)	p value^a^			
		N (%)	N (%)		d.f.^a^		
Sociodemographic Characteristics							
Gender							
Female	4595 (66.0%)	2319 (58.5%)	2276 (75.8%)	<.001	1		
Male	2368 (34.0%)	1642 (41.5%)	726 (24.2%)				
Age category							
60–64	1866 (26.8%)	1099 (27.8%)	767 (25.5%)	.027	4		
65–69	2085 (29.9%)	1193 (30.1%)	892 (29.7%)				
70–74	1067 (15.3%)	611 (15.4%)	456 (15.2%)				
75–79	1216 (17.5%)	678 (17.1%)	538 (17.9%)				
80+	727 (10.4%)	378 (9.5%)	349 (11.6%)				
Education							
<4 years	4594 (66.0%)	2451 (62.1%)	2143 (71.6%)	<.001	1		
≥4 years	2344 (33.7%)	1493 (37.9%)	851 (28.4%)				
Income							
Low income	4323 (62.1%)	2257 (59.3%)	2066 (70.5%)	<.001	1		
Higher income	2414 (34.7%)	1550 (40.7%)	864 (29.5%)				
Race/Ethnicity							
White	5862 (84.2%)	3370 (85.1%)	2492 (83.1%)	.070	2		
Afro-Brazilian	473 (6.8%)	255 (6.4%)	218 (7.3%)				
Other	625 (9.0%)	335 (8.5%)	290 (9.7%)				
Living arrangements							
Live with someone	5893 (84.6%)	3363 (85.2%)	2530 (84.3%)	.286	1		
Live alone	1056 (15.2%)	584 (14.8%)	472 (15.7%)				
Employed	940 (13.5%)	626 (15.9%)	314 (10.5%)	<.001	1		
Health Behaviors							
Physical activity (Yes)	2608 (37.5%)	1580 (40.2%)	1028 (34.3%)	<.001		1	
Use tobacco	1302 (18.7%)	763 (19.4%)	539 (18.0%)	.128		1	
Participate in social activities	2736 (39.3%)	1495 (37.8%)	1241 (41.3%)	.003		1	
Functional limitations and Health Conditions							
Limitations							
0 problems	4238 (60.9%)	2628 (66.4%)	1610 (53.6%)	<.001		2	
1–2 problems	2195 (31.5%)	1075 (27.2%)	1120 (37.3%)				
3+ problems	526 (7.6%)	254 (6.4%)	272 (9.1%)				
Hypertension	3410 (48.9%)	1688 (42.6%)	1722 (57.4%)	<.001		1	
Heart problems	1962 (28.2%)	894 (22.6%)	1068 (35.6%)	<.001		1	
Stroke	254 (3.6%)	138 (3.5%)	116 (3.9%)	.402		1	
Varicosities	1197 (17.2%)	475 (12.0%)	722 (24.1%)	<.001		1	
Diabetes	790 (11.3%)	397 (10.0%)	393 (13.1%)	<.001		1	
Back problem	2999 (43.1%)	1117 (28.2%)	1882 (62.7%)	<.001		1	
Osteoporosis	1047 (15.0%)	313 (7.9%)	734 (24.5%)	<.001		1	
Bronchitis	1917 (27.5%)	919 (23.2%)	998 (33.3%)	<.001		1	
Pneumonia	453 (6.5%)	195 (4.9%)	258 (8.6%)	<.001		1	
Urinary infection	1222 (17.5%)	516 (13.0%)	706 (23.5%)	<.001		1	
Renal problems	897 (12.9%)	317 (8.0%)	580 (19.3%)	<.001		1	
Dermatologic	724 (10.4%)	340 (8.6%)	384 (12.8%)	<.001		1	
Headaches	2248 (32.3%)	999 (25.4%)	1249 (41.6%)	<.001		1	
Gastrointestinal	1272 (18.3%)	533 (13.5%)	739 (24.6%)	<.001		1	
Depression							
0 symptoms	2438(35.0%)	1693(42.7%)	745(24.8%)	<.001		3	
1 symptom	1803(25.9%)	1040(26.3%)	763(25.4%)				
2 symptoms	1265(18.2%)	633(16.0%)	632(21.1%)				
3+ symptoms	1457(20.9%)	595(15.0%)	862(28.7%)				

a p-value and d.f. (degrees of freedom) based on chi square test; there are missing data for some variables.

Arthritis was reported by 43.1%, 76% of whom were women. Half of all women, compared to less than a third of men, reported arthritis. Report of arthritis increased slightly with age (age 60–64: 41%; age ≥80: 48%), and was more prevalent among persons with lower education and income. Those with arthritis were more likely to have a limitation, to report each of the health conditions examined including depressive symptomatology, and to be socially active, but were less likely to be physically active or employed.

Fully controlled analyses (Table 2) included all health conditions, but only the demographic, health behavior, and functional limitation variables that were significant in the analysis that included only these variables. Of the demographic characteristics, only gender and education were significantly associated with report of arthritis. The odds of self-reported arthritis was 63% greater in women than in men, 24% greater among persons age ≥80 than among persons age 60–64 (but this did not reach the p≤.01 level of significance), and 22% lower odds among those with ≥4 years of education.

**Table 2:** Concomitants of arthritis. Multivariable logistic regression for total sample.

	OR	(95% CI)	*p-*Value
Sociodemographic Characteristics			
Gender (female)	1.62	(1.44–1.82)	<0.001
Age category			
60–64	Reference		
65–69	1.05	(0.92–1.21)	0.474
70–74	1.03	(0.87–1.22)	0.715
75–79	1.02	(0.87–1.20)	0.804
80+	1.27	(1.05–1.55)	0.016
Education			
<4 years	Reference		
≥4 years	0.78	(0.70–0.88)	<0.001
Health Behaviors			
Participate in social activities	0.85	(0.74–0.98)	0.003
Functional limitations			
Number of Limitations			
0 limitations	Reference		
1–2 limitations	1.17	(1.04–1.31)	0.011
≥3 limitations	1.03	(0.83–1.28)	0.763
Health Conditions			
Hypertension	1.37	(1.22–1.53)	<0.001
Heart problems	1.10	(0.96–1.25)	0.180
Stroke	0.66	(0.49–0.87)	0.004
Varicosities	1.70	(1.48–1.95)	<0.001
Diabetes	0.98	(0.83–1.17)	0.855
Osteoporosis	2.74	(2.35–3.19)	<0.001
Bronchitis	1.28	(1.13–1.44)	<0.001
Pneumonia	1.06	(0.85–1.32)	0.597
Urinary infection	1.21	(1.05–1.40)	0.010
Renal problems	1.83	(1.55–2.15)	<0.001
Dermatologic	1.16	(0.98–1.37)	0.095
Headache	1.39	(1.23–1.56)	<0.001
Gastrointestinal	1.42	(1.24–1.662)	<0.001
Depression			
0 symptoms	Reference		
1 symptom	1.31	(1.13–1.51)	0.001
2 symptoms	1.43	(1.22–1.69)	<0.001
≥3 symptoms	1.58	(1.32–1.88)	<0.001

OR  =  Odds ratio.

CI  =  Confidence Interval.

Among health behaviors, only involvement in social activities was significant, the odds of social participation being reduced by 15% among those reporting arthritis (in contrast to greater participation in uncontrolled analysis). Although bivariate analysis indicated that arthritis was associated with functional limitations, the p≤.01 level was not reached in controlled analyses. Point estimates indicated a 17% increased odds of 1–2 (but not of more) limitations (p = 0.011).

The health problems with an increased odds of association with arthritis were hypertension (37% increased odds), varicosities (70%), osteoporosis (174%), bronchitis (28%), renal problems (83%), headache (39%), gastrointestinal disorders (42%), and depressive symptomatology (increasing from 31% greater odds with one symptom, to 58% with ≥3 symptoms). Heart disease, diabetes, pneumonia, and dermatologic disorders were not associated with arthritis in this sample. With stroke there was a 34% reduced odds of arthritis.

The final model, re-run with interaction terms for gender, indicated that while the odds of arthritis were twice as high in women compared to men, no significant gender differences regarding concomitants of arthritis were found.

## Discussion

There is considerable reasonably consistent information on the prevalence of arthritis in developed countries. There is less information from middle income countries, particularly on the older population, and findings on prevalence rates are less consistent. Further, although multimorbidity is common in older persons, studies on arthritis from Brazil (the location of the present study), have as yet reported on very few concomitant conditions [23]. The current study takes a broader approach, considering health behaviors and a wide range of chronic conditions relevant to older persons, in addition to demographic characteristics.

Primary findings from current data indicate that, in 1995, arthritis was a very common disorder in community residents ≥60 years of age in the southern state of Rio Grande do Sul. At 43.1% the prevalence rate was higher than the 1998 national prevalence rate of 37.5% [17], or local rate of 25.3% in the southern town of Bambui (a rate that became 51.9% when joint symptomatology was included) [23]. The manner of identifying arthritis varied across studies. It included self-report of treatment in the past six months (present study); acknowledging presence of the condition, presented in a list and without a time frame (PNAD 1998 survey) [27], self-report of medical diagnosis (PNAD 2003, 2008 surveys) [27]; a two-stage process: initial self-report that was accepted if doctor-diagnosis was reported, no time frame was set [23]; and acceptance of joint pain [23]. Finally, in the COPCORD studies (rate very low, and not given here because the number of older participants was small and included people age 55) [19], [28], [29], a medical diagnosis was required. Absence of agreement on diagnostic criteria, item phrasing, and time frame covered (from current to lifetime) makes comparison across studies hazardous, and helps to explain the broad range of prevalence rates found.

In the present study prevalence increased (but nonsignificantly) with age. Similarly, there was no age-associated increase in the Bambuí study [23]. The significance of increase with age was not reported for PNAD [18]. Arthritis was reported more frequently by women (in common with the major comparison studies), and by those with less education (agreeing with findings on joint symptoms in the Bambui study, but not with findings on arthritis). Arthritis was similarly present in the three racial/ethnic groups in the current study. The other studies from Brazil do not report on race/ethnicity.

The general association of arthritis with demographic characteristics was comparable to that in developed countries. There were, however, some differences in details. At 43%, the prevalence among those age 60 and over appears to be somewhat lower than findings in Australia (49% age 65–74, 50% age 75–84 [11]), the U.S. (age ≥65: 55% [38], and 50% [4], [39]–[40]), and Canada (1996: 39.7% age 65–74, 46.8% age 75+) [16], but higher than for Great Britain (age 65–74: men 12.7%, women 19.4%; age 75+ men: 13.1%, women 25.0% [12]). The differences across studies and countries may reflect differences in sample ages, in reference periods (ranging from current status to ‘ever told’), and in how arthritis is – or is not – defined. Our increased prevalence with age is comparable to that for Australia, and less steep than for Great Britain or the U.S. The U.S. samples, for instance, climb from 36.5% at age 55–64 to 57.1% at age ≥85 [2], [8], and from 40.2% (age 55–64) to 62.0% (age ≥85) [32], as compared with 41.1% (age 60–64) to 48.0% (age ≥80) in the current study, or from 34% (age 60–69) to 44.9% (age ≥80) in PNAD [18], i.e., an increase of ∼20% over 30+ years of age in the U.S., compared to ∼7%–∼11% over 20+ years of age for the Brazilian studies.

In common with other studies, self-reported arthritis was more frequent in older women than in older men (generally around 1.5∶1). This difference may be attributable to a change in estrogen level. Estrogens are known to influence bone metabolism [41] and a postmenopausal decrease in estrogen levels might result in an increasing risk of arthritis [42], [43]. Genetic and molecular studies have also shown that susceptibility to osteoarthritis (OA), by far the most common kind of arthritis, might be determined by genetic polymorphisms [44], and genes that operate differently in men, women, and in different ethnic populations [45], [46].

While rates were comparable, a larger proportion of Afro-Brazilians than White Brazilians reported limitations (51.8% vs. 45.3%), and their limitations were greater (12% vs. 8.7% needing help with ≥3 activities) (data not shown), findings comparable to the U.S. [30]. Controlled analysis found that of the health behaviors examined – physical activity, tobacco use, and involvement in social activities – only the latter remained significant. Absence of association with physical activity has been reported by others [47]. Although tailored physical activity can benefit arthritis sufferers, and physical activity can benefit all adults, the level of physical activity of both those with arthritis and those without arthritis has been found to be similarly low, with fewer arthritis sufferers meeting requirements for moderate or vigorous activity [47]. Reduced participation in social activities is, perhaps, to be expected, but is a reversal of that found in uncontrolled analysis, and is not supported by findings from the State of São Paulo, where social functioning, as measured by the SF-36, was not lower in older persons with arthritis than in those with none of the chronic conditions examined [48].

In bivariate analyses, a significant association was found between arthritis and functional limitations. This is not a new finding, but has been reported consistently. Previous studies, however, have rarely taken multimorbidity into account. When this is done (as here in controlled analyses), functional limitations no longer attained the necessary level of significance, agreeing with other reports that arthritis may be associated with mild, rather than severe functional limitations [25].

Studies reviewing comorbidities with arthritis have variously reported the presence of hypertension, cardiovascular and circulatory system disease, endocrine diseases (including diabetes), musculoskeletal diseases including osteoporosis, chronic pulmonary disease, infections, malignancies, central nervous system, skin and subcutaneous tissue, gastrointestinal disease, neurological and psychiatric disorders including depression, and dementia [11], [32]–[34], [49]. Whether all conditions occurred beyond chance is not clear. Some studies were well controlled [49], others less so.

In agreement, we found a higher than expected association with arthritis for hypertension, varicosities, osteoporosis, bronchitis, urinary infections, renal problems, headache, gastrointestinal disorders, and depressive symptomatology. Depressive symptomatology has been found to be common in chronic diseases [32], but the increased odds here of osteoporosis is suspect (as it was also in another study [48]). Similar proportions of men and women report osteoporosis, suggesting that some participants may have confused the two terms, which sound similar in Portuguese. The diversity of other conditions associated with arthritis, however, requires explanation. It is possible that there is an underlying connection through pain [48], with associated neuroendocrine, immunologic, and inflammatory dysfunction, and for some conditions through pharmacological intervention.

Because arthritis is a chronic painful condition, many of the health conditions associated with it may also be other chronic painful conditions. In support, significant associations have been reported between chronic headache and musculoskeletal symptoms [50]; pain and depression [51]; and pain, other health conditions present, and functional limitations [34]. In animal models of generalized pain, stress induced a switch of intracellular signaling in sensory neurons [52]. The enhanced pain response was associated with glucocorticoid and catecholamine activation at receptors located on sensory afferents. This animal model suggests that both the hypothalamic-pituitary-adrenal (HPA) axis and the autonomic nervous system are involved in stress-activating pain pathways, leading to chronic hyperalgesia. Stress releases corticotropin-releasing hormone from the hypothalamus, which stimulates the secretion of corticotropin and cortisol. Abnormal cortisol regulation may be associated with gastric ulcers, osteoporosis, hypertension, and depression, while long term low dose glucocorticoid use has been found to increase the prevalence of fractures, hypertension, myocardial infarction and serious infections [53]. Chronic pain pathways are affected by immune and inflammatory mechanisms [54], [55]. Cytokines such as tumor necrosis factor have a hyperalgesic effect [56]. Proinflammatory cytokines are produced in excess in people with asthma, rhinitis, headaches and possibly also in depression, osteoarthritis and rheumatoid arthritis [57]. They are also associated with inflammatory activation of the HPA axis [58].

Common pharmacological intervention in the form of nonsteroidal anti-inflammatory drugs (NSAIDs) to reduce pain in arthritis has long been recognized as increasing risk of adverse gastrointestinal, and renal events, although cardiovascular effects may be mixed. While we lack information on medications being taken, the ubiquity with which NSAIDs are prescribed suggests that their use may underlie the association found with gastrointestinal and renal disorders, as well as the increased odds of hypertension [59]–[61]. Unlike some previous studies, however, we found an absence of association with heart disorder (possibly due to the effects of aspirin), and reduced odds of stroke. There was also an absence of association with diabetes in controlled analysis. Since diabetes and arthritis share a common risk factor (obesity, of which unfortunately, we have no measure), this was unexpected. Absence of this association cannot be explained by treatments for rheumatoid arthritis, which have been found to be beneficial where diabetes is concerned [62]–[64], since rheumatoid arthritis is far less common than osteoarthritis. Thus, it is possible that pain, and treatment for pain, may help to explain the apparent diversity of conditions associated with arthritis, although other factors, including infection and genetics, should not be ignored.

### Limitations

Arthritis was self-reported, but in older persons self-report has been found to be reliable [65]. Our data may underestimate the prevalence of arthritis, since information was gathered only on arthritis of sufficient concern that medical treatment was sought in the previous six months. Mild arthritis, self-treated arthritis, or arthritis with no flares in the previous six months may not be represented. Association with other health conditions may also be under-estimated because information was only requested on conditions that had been brought to medical attention comparatively recently. Nevertheless, a wide range of conditions, which overlap with those previously reported, was found. Information on specific types of arthritis was not available, but such a level of detail is rarely requested in population-based surveys since response may not be credible [7]. It does, however, restrict our ability to compare our findings with that of others, who focus on specific conditions, and to explain our findings. We are undoubtedly missing symptomless cases that can be radiologically diagnosed [21]. Such cases, however, are likely to have a lesser public health impact. The extent to which current information can be generalized to the rest of Brazil is unclear. Our data were gathered in one of the richer states, and access to health care (and diagnosis of arthritis), may be different elsewhere. These data were gathered in 1995, and while a national health system was present at the time, there has since been increased access to care [66]. In consequence a similar survey carried out now might indicate a different prevalence of arthritis. Prevalence could also have increased since 1995 because of the rapid aging of the Brazilian population, and increased longevity. However, when we applied the self-reported arthritis prevalence rates from the current study to census–based population estimates for the State of Rio Grande do Sul for 1996 and 2010, we found little change between the two years. Between 1996 and 2010 there was an influx of younger men age 60–64 (who report the lowest rate of arthritis), which offset effects of increase in arthritis at the oldest ages. Although we have tried to restrict comparison across studies to comparable age groups, this has not always been feasible.

In summary, the prevalence and demographic characteristics of arthritis in older community residents in this southern part of Brazil has characteristics comparable to those found in developed countries. Most, but not all comorbid associations identified in previous studies were confirmed. In particular, we were able to determine the odds of these associations, odds which have rarely been presented. Further study is needed to confirm the present findings.
